# A Review of Pathophysiology, Clinical Features, and Management Options of COVID-19 Associated Coagulopathy

**DOI:** 10.1097/SHK.0000000000001680

**Published:** 2021-06-01

**Authors:** Julie Goswami, Taleen A. MacArthur, Meera Sridharan, Rajiv K. Pruthi, Robert D. McBane, Thomas E. Witzig, Myung S. Park

**Affiliations:** 1 Division of Trauma, Critical Care, and General Surgery, Department of Surgery, Mayo Clinic, 200 1^st^ St. SW, Rochester, MN, 55905; 2 Department of Hematology, Mayo Clinic, 200 1^st^ St. SW, Rochester, MN, 55905; 3 Division of Vascular Cardiology, Department of Cardiovascular Disease, Mayo Clinic, 200 1^st^ St. SW, Rochester, MN, 55905

**Keywords:** COVID-19, Coagulopathy, Coagulation, Thrombosis, Thromboembolism, DVT, Pulmonary Embolism

## Abstract

There is increasing evidence that novel coronavirus disease 2019 (COVID-19) leads to a significant coagulopathy, a phenomenon termed “COVID-19 associated coagulopathy”. COVID-19 has been associated with increased rates of both venous and arterial thromboembolic events, a source of significant morbidity and mortality in this disease. Further evidence suggests a link between the inflammatory response and coagulopathy associated with COVID-19. This presents a unique set of challenges for diagnosis, prevention, and treatment of thrombotic complications. In this review, we summarize and discuss the current literature on laboratory coagulation disruptions associated with COVID-19 and the clinical effects of thromboembolic events including pulmonary embolism (PE), deep vein thrombosis (DVT), peripheral arterial thrombosis, and acute ischemic stroke in COVID-19. Endothelial injury and augmented innate immune response are implicated in the development of diffuse macro- and microvascular thrombosis in COVID-19. The pathophysiology of COVID-19 associated coagulopathy is an important determinant of appropriate treatment and monitoring of these complications. We highlight the importance of diagnosis and management of dysregulated coagulation in COVID-19 in order to improve outcomes in COVID-19 patients with thromboembolic complications.

## Introduction:

As of September 2020, there have been over 31 million worldwide confirmed cases of coronavirus disease 2019 (COVID-19) caused by severe acute respiratory syndrome coronavirus-2 (SARS-CoV-2), with over 1 million confirmed deaths (1). In the United States alone, there have been over 7 million cases and over 200,000 deaths from the novel virus. In early retrospective studies, cohorts of COVID-19 patients in Wuhan, China were found to have disruptions in coagulation parameters including elevated D-dimer levels and prothrombin time (PT) (2, 3). These alterations in coagulation were predictive of a high mortality, with 15 of 22 (71.4%) of non-survivors meeting International Society of Thrombosis and Hemostasis (ISTH) criteria for disseminated intravascular coagulation (DIC) in comparison to just 1 of the 162 (0.6%) survivors in one early study (3). By March 2020, ISTH had published an algorithm for recognition and management of “coagulopathy in COVID-19” (4). Markers of coagulation disruption in COVID-19 currently under investigation include D-dimer levels, platelet count, Von Willebrand Factor (VWF), Factor VIII, thromboelastography (TEG), lupus anticoagulant (LAC), antiphospholipid antibodies (APLs), and fibrinogen.

In addition to the disturbances in laboratory markers of coagulation, COVID-19 infection has been associated with both venous and arterial thrombosis (5, 6). Autopsy studies of COVID-19 patients have shown both macro- and microvascular thrombosis (7, 8). The SARS-Cov2 virus uses angiotensin converting enzyme-2 (ACE-2) as its main receptor; this membrane protein is expressed in the blood vessels, lungs, heart, kidneys, and numerous other tissues. It has been hypothesized that SARS-CoV-2 binding to ACE-2 leads to local and systemic inflammatory response, endothelial injury, and an imbalance in pro- and anticoagulant signals, with resultant macro- and microvascular thrombosis (9). Ongoing mechanistically-driven and clinical research seeks to identify the most efficient pharmacologic targets for preventing and treating arterial and venous thrombosis associated with COVID-19 infection. We performed a systematic review of the literature to provide an update on derangements of coagulation laboratory assays, clinical manifestations of coagulopathy, and current recommendations for prevention and treatment of thrombotic complications in patients with COVID-19 infection.

## Methods:

**Figure 1:**
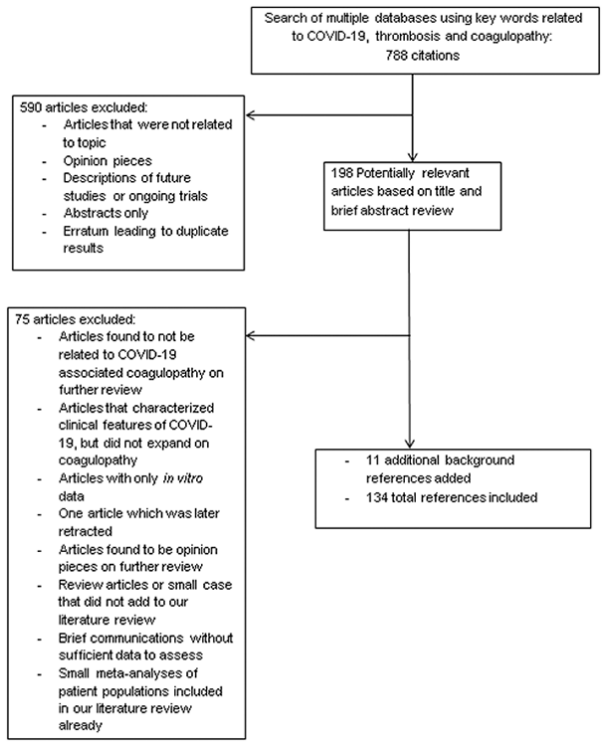
Description of Methods

This systematic review was carried out with the assistance of a Masters level librarian through Library Services at Mayo Clinic in Rochester, Minnesota. Multiple databases were interrogated including: EMBASE, MEDLINE, Scopus, Web of Science, Cochrane Database of Systematic Reviews, ACP Journal Club, Cochrane Central Register of Controlled Trials (CCTR), Cochrane Clinical Answers (CCA), Cochrane Methodology Register, Database of Abstracts of Reviews and Effects (DARE), Health Technology Assessment Database (HTA) and National Health Service Economic Evaluation Databases (NHSEED). The following keywords were utilized: COVID-19, coagulopathy, Coronavirus, COVID, SARS-COV-2, coagulation, endothelial, thrombosis, anticoagulation, thromboembolism, DVT, hypercoagulable, pulmonary embolism. We restricted our results to articles written or translated in English and the following article types: clinical trials, retrospective reviews, cohort studies, society guidelines, meta-analysis, case control studies and case series. This search yielded 788 individual citations, which were screened based on title and brief review of the abstract by two independent physician reviewers. From these, 590 articles were excluded. Articles were excluded at this stage for the following reasons: Publications that were clearly not relevant to the topic (for example, related to coagulopathy but not of COVID-19, articles related to other viral illnesses, articles related to other aspects of COVID-19), descriptions of future studies or ongoing clinical trials, erratum from studies yielding duplicate results, abstracts without full articles or abstracts in English with associated articles that were not translated, and opinion pieces. This resulted in 198 potentially relevant articles, which we reviewed in depth by two physician reviewers. From these, 75 articles were excluded. Reasons for exclusion at this stage were: Articles were found to not be pertinent on further review (for example, articles focused on other aspects of COVID-19), articles with only *in vitro* data that did not use any patient samples, articles that described clinical features of COVID-19, but did not expand on coagulopathy, one article that was later retracted, articles found to be opinion pieces on further review, brief communications without sufficient data to assess, review articles or small case series, small meta-analyses in which all or most of the articles were already assessed and incorporated in this review. The remaining 123 articles were then re-evaluated, verified for inclusion and summarized by two independent authors. We also reviewed the bibliographies of the 123 articles and performed thorough search for other relevant background references. This yielded 11 results, for a total of 134 cited references (Figure 1). Additionally, we present some unpublished data within this manuscript from our institution’s experience with COVID-19. Collection of this de-identified data was approved by the Mayo Clinic Institutional Review Board (IRB) and permission to utilize this data was provided to us by the study team.

## Laboratory Abnormalities in COVID-19 Patients:

### D-dimer:

Early work on COVID-19 associated coagulopathy noted consistently elevated levels of D-dimer, a marker of fibrin degradation by the action of plasmin on stabilized fibrin, in COVID-19 patients (2). Tang et al. showed that patients who died from COVID-19 had significantly greater D-dimer levels, pro-thrombin time (PT) and fibrinogen on admission as compared to survivors of COVID-19 infection (3). In another retrospective analysis of 343 patients from Wuhan, China, a D-dimer level ≥ 2.0 μg/dL was significantly associated with higher mortality (18% vs. 0.4%), with 92.3% sensitivity and 83.3% specificity (10). The dynamic association between D-dimer level and COVID-19 disease prognosis has been repeatedly demonstrated (11-13). Multiple studies also demonstrate a relationship between the trend of D-dimer levels and COVID-19 disease progression (14-17). Initial studies equated the elevation in D-dimer level with DIC, but ongoing work has suggested that COVID-19 associated coagulopathy is a unique entity (3, 4, 18). At our own institution’s special coagulation laboratory, we have measured soluble fibrin monomer levels (SFMC), a sensitive and specific marker of DIC, from patients who had elevated D-dimer levels. Of an initial cohort of 35 COVID-19 patients, D-dimer level ranged from 220-100,000 ng/mL (median 627 ng/mL), and 27 patients (77%) were found to have elevated D-dimer levels. Of these 27 patients, SFMC ranged from 5-1,100 mcg/mL (median 5 mcg/mL), and only 5 patients (18.5%) were found to have elevated SFMC. Additionally only 4 of the 35 (11%) patients included in this study were found to have ISTH DIC score ≥ 5 (cutoff for diagnosis of overt DIC). The low frequency of elevated SFMC and ISTH DIC score ≥ 5 suggests that the elevated D-dimer levels observed in COVID-19 does not represent overt DIC (unpublished Mayo Clinic data).

Elevated D-dimer level is a marker for venous thromboembolism (VTE) in the appropriate clinical setting. In COVID-19 patients, elevated D-dimer levels have been shown to be predictive of VTE development (19-21). A number of studies have sought to quantitatively define the association of D-dimer levels with VTE in COVID-19 (22). In a multicenter retrospective cohort study, Artifoni et al. showed that a D-dimer level < 1.0 μg/ml had a 95% negative predictive value (NPV) for venous thromboembolism (VTE), which encompasses deep vein thrombosis (DVT) and pulmonary embolism (PE), while a D-dimer of > 3.0 μg/mL had an 80% positive predictive value (PPV) for VTE (19). All patients in this study received appropriate weight-based pharmacologic VTE prophylaxis with enoxaparin. Elevated D-dimer levels also correlate with VTE in non-critically ill COVID-19 patients. In one study of 29 non-ICU patients, a D-dimer level of > 5,000 μg/dL was an independent predictor of PE (OR 3.77, 95% CI [1.18-12.16], p = 0.03) Similarly, in another study of 156 hospitalized patients, a D-dimer level of > 1,570 ng/mL was associated with a higher frequency of *asymptomatic* DVT (OR 9.1, 95% CI [1.1-70.1], PPV 19%), with a D-dimer level less than 1,570 ng/mL having a NPV of 97.5% for asymptomatic DVT (23, 24). Thus, D-dimer level potentially has a role in clinical decision making for patients with COVID-19 infection. It is worthwhile to note that different institutions and laboratories vary in unit of measurements for D-dimer levels, with the most common measurements being ng/mL, μg/ml, and μg/dL. The ISTH has recommended admission testing and monitoring of D-dimer, prothrombin time (PT), platelet count, and fibrinogen in all hospitalized COVID-19 patients (4). Further clinical and laboratory validation will be needed to better understand the roles of these labs in risk stratifying COVID-19 patients for VTE development (4).

### Thrombocytopenia:

Decreased platelet counts or thrombocytopenia appear to be uncommon, but associated with poor prognosis in COVID-19 (15, 25, 26). Multiple studies have shown an association between thrombocytopenia and mortality in COVID-19 (27). A retrospective study of 383 COVID-19 patients in Wuhan, China showed a threefold increased rate of mortality (30.9% vs. 8.9%) in patients with thrombocytopenia (platelet count < 125 x 10^9^/L) on admission as opposed to patients who did not have thrombocytopenia on admission. They additionally performed a Cox proportional hazards regression model that showed a 40% decrease in mortality associated with every 50 x 10^9^/L increased in platelet count (28). Similarly, Yang et al. showed that while thrombocytopenia (platelet count < 125 x 10^9^/L) was present in only 20.7% of 1,476 hospitalized COVID-19 patients, platelet nadir was associated with mortality, even when adjusted for age and gender (29). There are also reports of “delayed phase” thrombocytopenia. A retrospective study from Wuhan, China showed that 32 of 271 (11.8%) patients developed thrombocytopenia at 14 days after symptom onset, and the mean time from symptom onset to thrombocytopenia was 28.3 days (26). The majority of thrombocytopenic patients in this study were older with worse outcomes, including increased hospital length of stay and increased rate of mortality. It has been proposed that COVID-19 leads to thrombocytopenia through bone marrow suppression, either as a direct viral effect or from cytokine release (30-32). This is supported by evidence of impaired megakaryocyte maturation in bone marrow aspirates from thrombocytopenic COVID-19 patients (26). An alternate hypothesis is that thrombocytopenia occurs through a consumptive process of platelet destruction by immune complexes and generation of thrombi and microthrombi in the lungs. Although thrombocytopenia is certainly not present in the majority of COVID-19 patients, it is consistently linked to poor prognosis, at various stages in the disease process. Platelet count should thus be checked on admission and monitored in hospitalized COVID-19 patients (4).

### Von Willebrand Factor (VWF):

Studies have characterized a component of “endotheliopathy” in COVID-19 related coagulopathy. Endothelial injury by COVID-19 induced inflammation is thought to contribute to the hypercoagulable state (33, 34). A recent review identified disruption to endothelium and altered hemostasis as the key drivers in individual phenotypes of COVID-19 (35). In a case report, Escher et al showed markedly elevated plasma VWF antigen (VWF:Ag) and Factor VIII (FVIII) activity levels of 555%, 520% and 369% respectively in a critically ill COVID-19 positive patient on hospital day 21, indicative of endothelial cell injury (34). They also demonstrated a decrease in D-dimer levels and liberation from mechanical ventilation after starting heparin (34). Escher et al subsequently published a case series showing elevated levels of VWF, FVIII and fibrinogen in COVID-19 patients despite chemoprophylaxis (36). Persistently elevated levels of VWF have also been shown in both critically ill and non-critically ill COVID-19 patients (33, 37, 38). Additionally, there is a case series showing a relative reduction in levels of ADAMTS13 in COVID-19, further pointing to the potential role of endothelial injury in these patients (38). Although measurement of VWF level and activity are not common practice in the clinical setting, these data support that the coagulopathy of COVID-19 results, in part, from widespread endothelial injury.

### Antiphospholipid Antibodies:

More recently, there has been evidence that various anti-phospholipid antibodies (APLs) are present in COVID-19 patients. Several studies have reported presence (45-85%) of lupus anticoagulant (LAC) in the blood of critically ill COVID-19 patients (37, 39-41). Elevation of LAC is not typically measured during acute illness, and expression has been transient and variable in COVID-19 patients, with no definitive association to thrombotic complications (42). This effect may be due to non-specific assay interference with C-reactive protein (CRP) (43). Although the clinical significance of LAC in patients with COVID-19 remains uncertain, high LAC titers are associated with prolonged activated partial thromboplastin time (aPTT), which can be problematic when using aPTT for monitoring of unfractionated heparin therapy (40). As such, several authors advocate using anti-Xa levels to guide heparin treatment in COVID-19 patients (40, 44).

In a cohort of 74 mechanically ventilated COVID-19 patients, anti-cardiolipin IgG and/or IgM and/or anti-B2-glycoprotein-I (anti-B2-GP1) IgG were detected in 9 patients, but did not correlate with thrombosis or coagulopathy (42). In a group of 56 COVID-19 patients, although 45 patients were positive for LAC, anti-cardiolipin or anti-B2-GP1, IgG and IgM were detected in only 5/45 patients (10%) (41). In another study of 66 critically ill COVID-19 patients in China, APLs were present in 31 (47%) patients, with IgG anti-B2-GP1 identified in 19 (28.8%) of these 66 patients (39). This study found that 5 of 15 patients (33%) who had multiple elevated APLs suffered ischemic stroke, compared to 0/25 with normal levels and 0/16 with elevation of a single APL (39). Weakly positive anti-cardiolipin and anti-B2-GP1 IgM (no IgG) were also described in 2 of 24 (8.3%) of patients in a cohort of COVID-19 patients in Madrid with confirmed VTE (45). IgA type APLs, which are not thought to play a role in pathogenesis, have also been described in COVID-19 patients (39).

APLs have been associated with other systemic viral illnesses but typically are not associated with an elevated thrombotic risk (46). COVID-19 infection is associated with an overall hypercoagulable state, but it is unclear whether the presence of APLs contributes to this coagulopathy or represents cross-reactivity between the virus and host cell receptors. It has been proposed that host B2-GP1 domains are exposed as a result of SARS-CoV-2 induced endothelial cell injury and give rise to cross-reactive anti-B2-GP1 antibodies or anti-cardiolipin antibodies (42). Further investigation is needed to elucidate the potential role of APLs within the schematic of COVID-19 associated coagulopathy. Current ISTH guidelines advocate for testing and diagnosis of anti-phospholipid antibody syndrome, if clinically indicated, with confirmatory testing 12 weeks following initial detection, even in patients with multiple positive APLs (47).

### Thromboelastography and COVID-19 Coagulopathy:

Thromboelastography (TEG) is a dynamic measure of the viscoelastic properties of coagulation and is increasingly employed in critical care, emergency, and surgical settings, particularly to guide plasma and platelet transfusion in bleeding patients. Several studies have found COVID-19 patients to be hypercoagulable by TEG or rotational thromboelastometry (ROTEM). In a series of 21 COVID-19 ICU patients, 90% (n=19) demonstrated hypercoagulability based on ICU admission TEG parameters (48). In a cohort of 24 intubated COVID-19 patients with a total of 30 TEGs drawn, half the measurements (n=15) had shortened R-times, 90% (n=27) had shortened K-times, and 87% (n=26) had increased maximum amplitude (MA) indicating more rapid clot formation and increased clot strength (49). Other small series of COVID-19 patients admitted to the ICU had similar findings in which ROTEM measurements demonstrated maximum clot firmness (MCF) above the normal range, along with elevated levels of D-dimers and fibrinogen (18, 50). Authors postulate that their findings indicate a widespread shutdown of fibrinolysis, with elevated D-dimer levels driven by local fibrinolysis in the lungs (18). In a cohort of 44 ICU patients, 57% showed evidence of complete “fibrinolysis shutdown” with elevated D-dimer levels and a Ly30 of 0% (51). This group went on to describe a 50% VTE rate and 80% rate of new hemodialysis requirement in patients with both D-dimer level > 2,600 ng/mL and Ly30 of 0% (51). Another small retrospective series of 25 COVID-19 ICU patients demonstrated “fibrinolysis shutdown” (defined by the authors as EXTEM maximum lysis of <3.5%) in 11/21 patients, with 8 of those 11 patients going on to develop a thrombotic event (52). Similarly, others have found elevated D-dimer levels to correlate with specific abnormal parameters on TEG (53). There are others who believe that TEG and ROTEM may not be well suited for identifying decreased fibrinolytic activity, particularly as it may be possible that large amounts of deposited fibrin in COVID-19 patients may be due to overwhelming physiologic response (54). As such, TEG and/or ROTEM, when interpreted in the context of other lab parameters, can be a helpful adjunct test both in understanding an individual’s coagulome and stratifying VTE risk.

## COVID-19 Associated Coagulopathy Leads to Increased Venous and Arterial Thrombosis

The clinical phenotype of COVID-19 associated coagulopathy includes macro- and microvascular arterial and venous thrombosis. Evidence of increased thrombotic complications in COVID-19 was first shown by Klok et al. who reviewed 184 patients with proven COVID-19 associated pneumonia from three intensive care units (ICUs.) They found a 31% total rate of venous and arterial thrombotic complications, with 81% of these being PE (5). The only independent predictors of thrombotic events in this initial study were age and coagulopathy defined as pro-thrombin time (PT) prolongation > 3s or activated partial thromboplastin time (aPTT) prolongation > 5s from the upper limits of normal (5). In a follow up study, patients with venous and arterial thrombotic complications related to COVID-19 were found to have greater all-cause mortality (55). Additional single-center studies have shown varying rates of venous and arterial thromboembolic events associated with COVID-19. A single-center retrospective study of 92 ICU patients found that 37 of the 92 (40%) had thrombotic complications, with the majority (n=19) being PEs (6). However, this study also found that 19 patients (21%) had hemorrhagic complications. Of these 19 patients, 16 were on therapeutic anticoagulation with 8 started on therapeutic anticoagulation in the absence of a confirmed thrombotic event (6). Their work emphasizes the importance of carefully considering the risks of therapeutic anticoagulation. An additional retrospective review of 362 hospitalized patients revealed a 7.7% (n=22) rate of arterial and venous thromboembolic events, higher in ICU patients than general care patients, with 57% (n=16) of these being VTE (56).

There have been a number of publications studying VTE specifically in critically ill cohorts of COVID-19 patients. In a retrospective single-center study of 75 ICU patients with COVID-19 associated pneumonia, 25 (33.3%) were found to have thromboembolic complications, with all patients requiring high doses of heparin to achieve therapeutic state (57). In a cohort of 91 critically ill COVID-19 patients, 24 (26%) developed VTE (58). VTE is a significant problem in non-critically ill patents as well. In a retrospective single-center study of 289 consecutively admitted COVID-19 patients on the general care floor, 49 patients (17%) were found to have VTEs, with 42 of these being PE. Patients who developed VTE were more likely to require transfer to ICU, had greater length of stay, had lower hemoglobin levels at discharge, and had greater peak D-dimer levels than those who did not develop VTE. On multivariable analysis, time from symptom onset to admission, international medical prevention registry on venous thromboembolism (IMPROVE) score, leukocyte count at admission, and lack of pharmacologic VTE prophylaxis, were independent predictors of VTE (59).

Al-Samkari et al. performed a multicenter, retrospective study of 400 patients and found a VTE rate of 4.8% (in 19 patients), arterial thrombosis rate of 2.8% (in 11 patients), and an additional 8 patients with recurrent clotting of dialysis circuits, for a total thrombotic complication rate of 9.5%. They additionally identified 21 bleeding complications in 19 patients (25). For each of these endpoints, the rate was greater in ICU patients compared with patients admitted to the general care floor (25). A multicenter prospective study demonstrated a significantly greater rate of arterial and venous thromboembolic events, particularly PE, for patients with COVID-19 related acute respiratory distress syndrome (ARDS) vs. matched patients with non-COVID-19 related ARDS (9 of 77 [11.7%] vs. 7 of 145 [4.8%]) (37). At our own institution, we have collected data from a cohort of 105 hospitalized COVID-19 patients receiving pharmacologic VTE prophylaxis and found a total of 6 (5.9%) arterial and venous thromboembolic events (unpublished Mayo Clinic data), including 3 VTEs, 2 myocardial infarctions, and one ischemic stroke. Additional thrombotic complications reported in literature have been central line associated thrombosis, clotting of continuous renal replacement therapy circuits, and veno-venous extracorporeal membrane oxygenation (ECMO) circuit complications (25, 55). Recently, two meta-analyses have estimated overall VTE risk in COVID-19 patients receiving pharmacologic prophylaxis at 23.9% and 31% (60, 61). It does appear that the elevated VTE risk may continue even after hospital discharge. In a prospective multicenter study of 1,877 discharged COVID-19 patients, the rate of VTE was determined to be 4.8 per 1,000 discharges (with 42 day follow up). The rate from the prior year was 3.1 per 1,000 discharges but this was not a statistically significant difference (62). Further studies of ambulatory and post-discharge COVID-19 patients are needed to better elucidate the potential VTE risks to these patients even outside of the hospital setting. For hospitalized patients, both on the general wards and in the ICU, vigilant monitoring for VTE and timely prophylaxis are recommended.

### Pulmonary Embolism:

A number of studies have focused on incidence and factors predictive of PE for COVID-19 patients in various clinical settings (Table 2). An initial case series of 20 patients revealed that 8 (40%) had findings of peripheral, multifocal PE on CT angiogram (CTA), along with elevated D-dimer and greater Revised Geneva Score (63). A retrospective study of 72 *non-hospitalized* COVID-19 patients referred to the emergency room for imaging demonstrated an 18% (n=13) rate of PE (64). In this series, patients with PE were found to be significantly older and have significantly greater D-dimer levels than those without PE (64). A larger, single-center study of 1,477 patients admitted with COVID-19 revealed that of the 214 who underwent CT angiography (CTA), 80 were identified to have PEs, thus a 5.9% rate in total patients included (65). Patients with PEs were more likely to have required ICU care and had greater D-dimer and C-reactive protein (CRP) levels in comparison to patients who had negative imaging (65). A retrospective multicenter study of 1,240 COVID-19 patients revealed an 8.3% (n=103) rate of PE (66). Multivariable analysis revealed male sex, administration of anticoagulation at prophylactic or therapeutic dose, elevated CRP, and days from symptom onset to hospitalization to be independent predictors of PE (66). An additional retrospective multicenter study of 135 COVID-19 patients, all of whom underwent chest CTA revealed 32 PEs (24%). Subgroup analysis showed that 12 of 24 patients admitted to the ICU (50%) developed PE. In comparison to patients who did not develop PE, patients who developed PE in this cohort had greater D-dimer levels and were more likely to require ICU admission with mechanical ventilation (67).

Patients requiring ICU care have been the focus of a number of studies on PE in COVID-19. A study of 106 consecutively admitted *critically ill* patients with COVID-19 revealed 22 (20.6%) patients developed PE (68). Historical controls from the prior year with similar illness severity were found to have a 6.1% (12 of 196) rate of PE (68). A study of 40 critically ill COVID-19 patients at a single center, all of whom required mechanical ventilation and underwent CTA, found 13 PEs (40% rate). Of these 40 patients, 22 received standard dose pharmacologic prophylaxis (4000 IU subcutaneous enoxaparin daily), and of these, 11 (50%) developed PE. The other 18 patients received either high dose pharmacologic prophylaxis (4000 IU enoxaparin twice a day) or therapeutic unfractionated heparin (1500-2200 IU/hr) and only 2 (11%) developed PE. The authors of this study advocate consideration of higher dose regimens in COVID-19 patients (69). Mak et al. were the first to study a cohort of COVID-19 patients requiring ECMO for refractory hypoxemic respiratory failure. Of the 51 patients included in this study, 18 (35%) were found to have evidence of pulmonary artery thrombus, and 28 (54%) were found to have evidence of some pulmonary ischemic changes, typically peripheral wedge shaped low-attenuation areas (70). Meanwhile, looking at 452 consecutively admitted *non-critically ill* COVID-19 patients at a single-center, Mestre-Gomez et al. found 29 (6.4%) patients with PE despite adequate pharmacologic prophylaxis, with D-dimer > 5,000 μg/dl being the only significant predictor of PE on multivariable analysis (23). Their data also suggest that hyperlipidemia requiring statin use may actually have a potentially protective role in the development of PE in COVID-19 patients (23). This heterogeneous group of studies (case series, retrospective single and multi-center studies) suggests the rate of PE in COVID-19 patients ranges from 5.9%-40%. Important clinical characteristics associated with PE appear to be elevated D-dimer levels, possibly the trend of D-dimer level, and need for ICU care.

### Deep Vein Thrombosis:

In the early months of COVID-19, a number of retrospective studies from Wuhan, China showed that hospitalized COVID-19 patients had a DVT rate ranging from 25 - 46% (20, 71, 72). In multiple studies, increased D-dimer levels were significantly associated with DVT (14, 20, 28). In another early study, 49 COVID-19 patients presenting to the ER with signs and symptoms of DVT underwent duplex of lower extremities. When they were compared to 141 COVID-19 negative patients presenting with similar signs and symptoms, there was a 6.1% rate of DVT in COVID-19 patients compared to a 3.5% rate patients without COVID-19 (73). After these initial studies, a number of groups published work in cohorts of COVID-19 patients who underwent screening duplex for DVT. A prospective study of 156 patients with COVID-19 and elevated D-dimer revealed a 14.7% (n=23) rate of *asymptomatic* DVT but multivariable analysis revealed significantly greater D-dimer levels in patients who developed DVT (24). A similar prospective, single center study of 84 consecutively admitted non-ICU patients with COVID-19, all of whom received pharmacologic prophylaxis (enoxaparin or fondaparinux) and underwent screening lower extremity duplex, found 10 (11.9%) patients with DVT (74). Elevated D-dimer levels, prior or active cancer history, and need for high-flow nasal cannula or non-invasive ventilation all were significantly associated with development of DVT (74). In 198 hospitalized COVID-19 patients followed over time, the prevalence of DVT at 7, 14, and 21 days was 16%, 33%, and 42% (75). This series found higher rates of DVT in patients admitted to the ICU vs. those admitted to the general care floor, and also showed an association of DVT with mortality (75).

Just as with PE and other VTEs, there appears to be a greater rate of DVT in critically ill COVID-19 patients, who have been the focus of numerous studies. In a retrospective review of 45 critically ill COVID-19 patients who received enoxaparin for prophylaxis, required intubation and mechanical ventilation, and underwent lower extremity ultrasound, DVTs were identified in 19 of 42 (42.2%) patients (76). In this cohort, patients who developed DVT were more likely to have greater D-dimer levels and ultrasound performed earlier in hospitalization, though the indications for ultrasound are not elaborated on in the study (76). In another prospective single-center study, Voicu et al. were able to perform screening lower extremity duplex in 56 consecutively admitted critically ill COVID-19 patients at the time of admission and again after 7 days. They found a total of 26 (46% rate) DVTs and patients with DVT were once again found to have greater D-dimer than those who did not develop DVT (77). While the reported rate of DVT associated with COVID-19 has been widely varied (6-46%)(Table 2), DVT is consistently associated with elevated D-dimer levels and ICU admission status.

### Cerebrovascular disease:

Acute ischemic stroke is associated with COVID-19 and has been reported as a presenting symptom (78). In an early single-center retrospective study of 219 patients in Wuhan, China, 10 (4.6%) developed acute ischemic stroke (79). Clinical features associated with stroke were age, disease severity, cardiovascular risk factors, elevated CRP and D-dimer levels (79). Merkler et al. performed a retrospective study of 2136 patients presenting to the ER or admitted with COVID-19 vs. 1516 patients with influenza (80). There was a statistically significant increase in acute ischemic stroke (1.5% vs. 0.2%) in patients with COVID-19 as compared to those with influenza, and this difference remained significant when adjusted for age, sex, and race (80). Multiple case series have demonstrated that ischemic stroke can be the presenting symptom of COVID-19 with high rate of mortality and residual morbidity, including paralysis and aphasia (81).

A multicenter study of 3,556 patients hospitalized with COVID-19, found acute ischemic strokes that were verified by imaging in 32 (0.9%) patients (82). In comparison to contemporary controls without COVID-19, who developed acute ischemic stroke, these patients had greater frequency of “cryptogenic” strokes, greater initial NIH stroke scale, greater peak D-dimer level, and higher mortality (82). Sweid et.al performed a retrospective case series of 22 patients with COVID-19 who developed acute stroke, largely thrombotic or embolic in nature (83). Many of these patients had involvement of both arterial and venous vasculature and multi-vessel involvement. Of the 16 of 22 patients who required mechanical thrombectomy, many of the procedures were described as complicated due to clot burden and consistency (83). Finally, a meta-analysis of 39 studies with a total of 135 cases of acute ischemic stroke in patients with COVID-19 describes a pooled frequency of 1.2% (84). Laboratory and imaging features associated with the occurrence of acute ischemic stroke include elevated D-dimer and fibrinogen, presence of antiphospholipid antibodies, and large vessel thrombosis, embolism, or stenosis (84). Acute stroke was associated with 38% overall mortality (84).

With ongoing studies, it is becoming evident that COVID-19 patients are also prone to intracerebral hemorrhage (ICH) due to disruptions in the microvascular circulation. In a retrospective multicenter cohort with diverse patient population (52% female, 68% black), Rothstein et al. identified 20 ischemic strokes (2.4% rate) in 844 patients, but also found 8 patients with hemorrhagic stroke (0.9%) (85). Of 4,131 COVID-19 patients admitted to three centers, 115 underwent brain MRI and 25 (overall rate 0.6%) had findings consistent with leukoencephalopathy or cerebral micro-bleeds. In comparison to patients who had negative MRIs, these 25 patients had lower Glasgow Coma Scale (median 6 vs. 14), imaging later in hospital course, greater peak D-dimer level, lower nadir platelet count, greater international normalized ratio (INR), longer duration of ventilator support, longer hospitalization, and higher mortality (20% vs. 9%) (86). A case series of 5 COVID-19 patients with ICH showed that 4 of these were in lobar territories. The authors comment that typically lobar ICH only represents 15 - 30% of cases and is associated with vascular anomalies; however none of the 5 patients in this series had identifiable vascular anomalies. The authors postulate that mechanisms contributing to ICH in COVID-19 may include direct and indirect endothelial injury or disruption of renin-angiotensin system (87). After identifying 2 COVID-19 patients on ECMO who suffered fatal frontal intraparenchymal hemorrhage in the absence of laboratory derangements, Heman-Ackah and colleagues have advocated for routine imaging in patients in whom routine neurologic exams are unreliable due to sedation and paralysis (88). Though these studies describe < 5% rate of acute cerebrovascular events in COVID-19 patients, these events are characterized by unique pathologic features and high mortality, and there is increasing evidence of association between COVID-19 and ICH. It is clear that imaging studies are necessary to determine treatment course, but ongoing prospective studies are necessary to determine individual risk stratification and need for imaging.

### Peripheral Arterial Thromboembolism:

In addition to acute ischemic strokes, COVID-19 has been associated with peripheral arterial disease. As with ischemic stroke, acute lower extremity arterial occlusion has been seen as a presenting symptom of COVID-19 in patients with no prior history of hypercoagulability or peripheral vascular disease (89). A single-center study was published after the authors noted an increase in the number of procedures necessitated by acute limb ischemia; from January through March 2020, 16.3% (23/141) of the vascular procedures were done for acute limb ischemia, and 87% (20/23) were positive for COVID-19 (90). As a comparison, only 1.8% (3/163) vascular interventions performed in the previous year had been done for acute limb ischemia (90). This phenomenon was further characterized in a propensity matched study of 16 COVID-19 positive and 32 COVID-19 negative patients who underwent lower extremity CT angiography (CTA) after presenting with symptoms of acute limb ischemia (91). All 16 of the COVID-19 patients were found to have evidence of at least one level of arterial thrombosis while 69% of COVID-19 negative patients had clot burden. Additionally, the COVID-19 patients had greater “clot score” by three different measures (91). While the rate of surgical intervention did not differ between the two groups, the patients with COVID-19 had a 38% limb amputation rate (compared to 3% for COVID-19 negative patients) and a 25% mortality (compared to 3% for COVID-19 negative patients) (91). These initial studies suggest that peripheral arterial thromboembolism contributes to increased morbidity and mortality in COVID-19 patients.

### Post-Mortem Studies:

Autopsy reports confirm the increased frequency of thromboembolic events observed in patients with COVID-19. In a series of the first 80 autopsies performed for patients who died of COVID-19 in Hamburg, Germany, 32 were found to have DVTs including 17 with concurrent PE (7). Similarly, a case series of 10 autopsies done in Austria showed some degree of small and medium vessel pulmonary thrombosis in all patients (92). Further post-mortem clinicopathologic examinations have revealed a prominence of diffuse alveolar hemorrhage, severe pulmonary capillary congestion, and peripheral and alveolar microvessel thrombosis in deceased COVID-19 patients (93, 94). In a case series of 7 autopsies, platelet rich microthrombi were found not only in the pulmonary vasculature but also in hepatic, cardiac, and renal vasculature (8). This study also suggested greater than usual numbers of megakaryocytes in pulmonary and cardiac tissues (8). Another post-mortem case series showed co-localization of SARS-CoV2 envelope proteins with C4b and C5b-9 in pulmonary microvasculature along with alveolar microvessel thrombosis, suggesting a role of complement in COVID-19 associated coagulopathy (95). Microvessel thrombi have also recently been demonstrated in vivo using hand held video capillaroscopy of sublingual vessels in a series of 13 critically ill COVID-19 patients requiring mechanical ventilation (96).

A number of novel techniques have been used in autopsy studies of COVID-19 patients. Of 86 consecutive COVID-19 deaths in one series, 68 were described as unexpected, in comparison to 70 of 334 deaths at the same center the year prior. Of these, 64 of 68 patients underwent post mortem computed tomography and 15 (23%) were found to have proximal PE, in comparison to just 5 of 70 (7.1%) from the prior year (97). Ultrasound-guided minimally invasive autopsy was performed in the first 10 fatal cases of COVID-19 in Brazil and demonstrated all patients to have diffuse alveolar damage with high density of megakaryocytes and 8 of the 10 patients to have fibrinous thrombi in alveolar arterioles (98). To investigate the interactions between inflammatory response and pulmonary microangiopathy, Li et al. used high resolution microscopy to create 3D reconstructions of lung tissue samples from patients who died from COVID-19 infection (99). Their 3D reconstructions revealed extensive small vessel thrombosis, distinct from diffuse alveolar hemorrhage, mature megakaryocytes within the small vessels of the lung, and fibrin deposition with inflammatory cell attachment, which were much more extensive than appreciated in 2D histology (99). Together, the emerging evidence from post-mortem studies suggests that COVID-19 is a disease of pulmonary microangiopathy with involvement of inflammation and coagulation.

## Innate Immune Response and Endothelial Injury Contribute to COVID-19 Associated Coagulopathy

**Figure 2:**
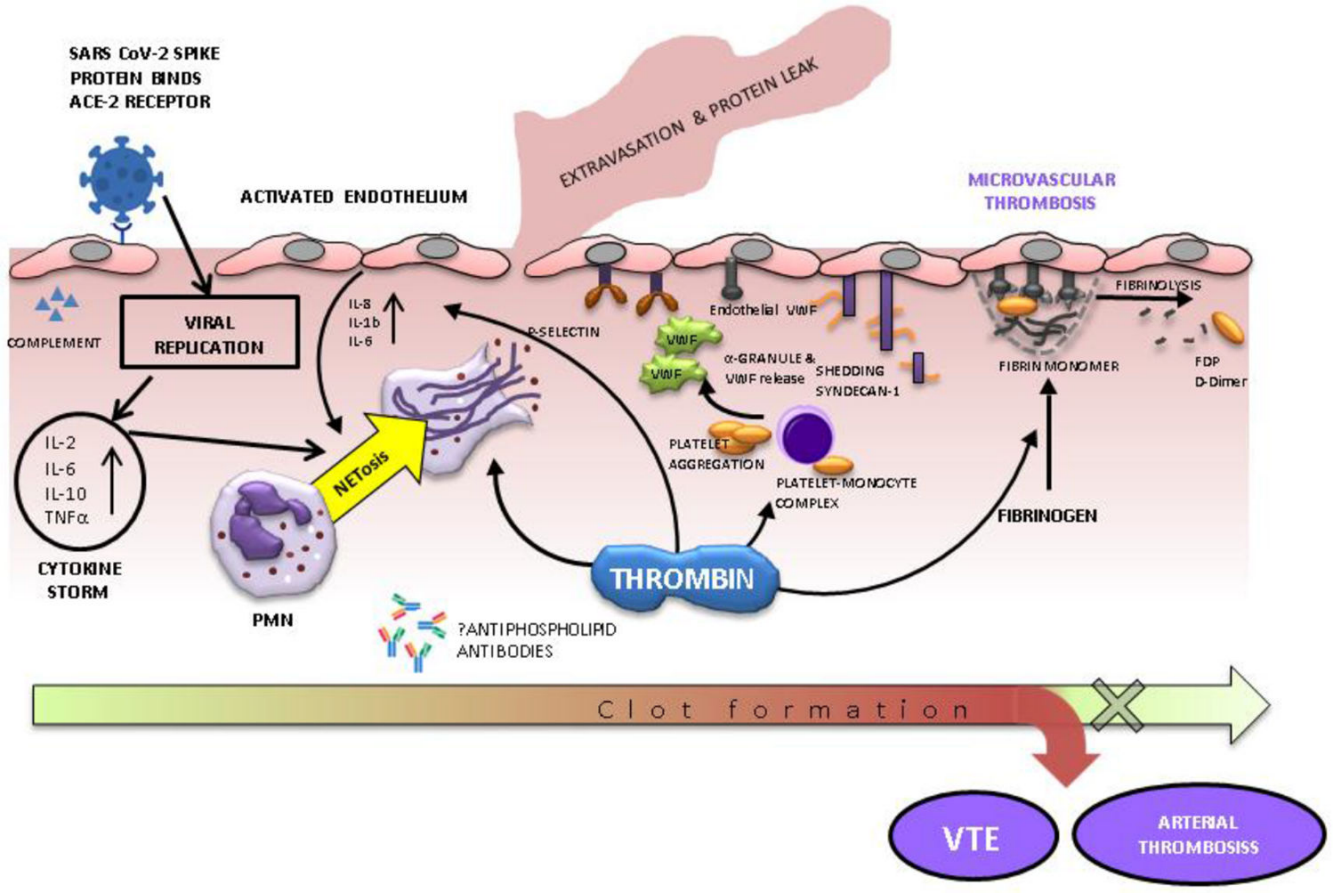
Proposed mechanisms by which SARS Cov-2 infection leads to micro- and macrovascular thrombosis

The complex interactions between inflammation and coagulation have been implicated in sepsis, DIC, ischemia-reperfusion, trauma-induced coagulopathy, and many other systemic disorders (44). There is mounting evidence for an inflammatory component of COVID-19 associated coagulopathy, with one study even showing elevated leukocyte count on admission to be an independent predictor of VTE (59). Proposed contributing factors include endothelial injury, cytokine storm, complement activation, particularly the alternative pathway which has recently been shown to trigger a hypercoagulable state, and formation of neutrophil extracellular traps (Figure 2) (100, 101). Fogarty et.al compared 33 critically ill and non-survivors of COVID-19 to 50 non-critically ill survivors (102). The 33 patients who required ICU stay or died had comparatively greater CRP, D-dimer, and fibrinogen at day 1 and 4, suggesting both inflammatory and coagulation disruption correlate with poor prognosis (102). The Global COVID-19 Thrombosis Collaborative Group describes “thromboinflammation” as an area of ongoing interest for identifying pharmacologic targets to prevent thrombosis (44). Elevated levels of cytokines including IL-2, IL-6, IL-10, TNF-alpha, and diffuse alveolar inflammation with fibrin deposition form the basis for their investigation (44). It is also thought that cytokine release may be a driver of direct and systemic damage related to COVID-19 (103). Immunomodulators targeting the complement cascade, JAK-kinase, or IL-6, with anti-thrombotic properties are potential preventative options in patients who have not yet developed thrombosis (44).

Endothelial injury may be an inciting insult for inflammatory mediated thrombosis (Figure 2). Case series of critically ill COVID-19 patients have shown persistent severe elevation in VWF, fibrinogen, and factor VII, all of which are suggestive of endothelial injury (34, 36). However, unlike patients with TTP or DIC, these patients have normal platelet counts and many have normal ADAM-TS13 activity (36). This has been further investigated with other markers of endothelial injury. In a single-center study, 40 ICU patients with COVID-19 were found to have significantly greater levels of soluble P-selectin, a marker of endothelial cell and platelet activation, in comparison to 10 COVID-19 positive patients who did not require ICU care (33). Furthermore, the authors found that levels of thrombomodulin, another marker of endothelial cell activation, were associated with in-hospital mortality (33). In another single-center study, endothelial cell adhesion markers, including vascular cell adhesion molecule-1 (VCAM-1), intercellular adhesion molecule-1 (ICAM-1), and vascular adhesion protein-1 (VAP-1), were elevated in 39 COVID-19 patients in comparison to 32 COVID-19 negative controls, and levels of elevation had a correlation to disease severity (104). Based on their findings, the author proposed that COVID-19 infection leads to release of pro-inflammatory cytokines which cause endothelial injury. Endothelial injury subsequently leads to altered expression of pro and anti-thrombotic factors and thrombosis (104). An alternate proposed theory is that SARS-CoV-2 viral infection leads to damage of the endothelial cell plasma membrane resulting in oxidative stress and release of reactive oxygen species which prime vessels for thrombosis (105). Interestingly, circulating endothelial cells (CECs) were found to be elevated in 66 COVID-19 patients when compared to 30 COVID-19 negative patients, but treatment with therapeutic anticoagulation led to significant decrease in CECs (106). Ongoing studies are necessary to further define the connections between endotheliopathy and coagulopathy in COVID-19.

Monocytes and neutrophils are two innate immune cells of particular interest in “thromboinflammation” or “immunothrombosis” associated with COVID-19. A recent prospective study of 35 patients with severe COVID-19 infections found that markers of platelet activation (P-selectin and thromboxane B2) correlates with elevated CRP (32). The group went on to perform immunofluorescence assays that showed increased platelet-monocyte aggregates in patients with severe COVID-19 infection in comparison to mild and asymptomatic patients and COVID-19 negative controls (32). They further discovered that tissue factor (TF), a major activator of the extrinsic pathway of coagulation, was expressed by monocytes, especially those monocytes in complex with platelets (32). This suggests that the interaction between cell types was necessary to induce coagulation cascade. Ex vivo studies revealed that platelets isolated from COVID-19 patients were able to induce TF expression by monocytes (32). Additionally, plasma from COVID-19 patients incubated with platelets isolated from healthy controls resulted in platelet activation, suggesting that circulating markers can lead to platelet activation (32). Their experiments suggest bi-directional signaling between platelets and monocyte TF expression leading to activated inflammation and hypercoagulability in COVID-19.

Neutrophils, known as first responders in innate immunity, have been well described in the last two decades to form neutrophil extracellular traps (NETs) by which they extrude chromatin studded with histones and other damage-associated molecular patterns or DAMPs. NETs have since been described to play a role in sepsis, autoimmune disorders, cancer, and a plethora of other systemic inflammatory disorders (107-109). In particular NETs contribute to micro- and macrovascular thrombi in a number of models (109, 110). The first pilot study looking at NETs in COVID-19 was performed at a single-center comparing 50 hospitalized COVID-19 patients with 30 healthy controls (111). Markers of NET formation, including cell free DNA, myeloperoxidase-DNA complexes (MPO-DNA), and citrullinated histone-3 (citH3), were elevated in the COVID-19 patients in comparison to healthy controls (111). Furthermore, there was an increasing trend of all three markers of NET formation with worsening oxygenation (111). In vitro studies combining serum from COVID-19 patients with neutrophils isolated from healthy controls, led to NET formation, suggesting that circulating markers are able to induce NET formation (111). Several groups have confirmed elevated levels of circulating NET degradation products including myeloperoxidase-DNA (MPO) with autopsy studies showing NETs present in microvasculature of lungs, liver, and kidney (101, 112, 113). In a study of 33 COVID-19 patients with 17 matched healthy controls, the COVID-19 patients were identified to have greater circulating platelet-neutrophil aggregates by flow cytometry. The 3 COVID-19 patients who died in this cohort were found to have co-localization of NETs with platelets in lung tissue (101). At our institution we have recently begun collecting samples from COVID-19 patients to assess for markers of NET formation, complement activation, and fibrinolysis. This data may pave the way to individualized VTE risk stratification in COVID-19 patients.

It has recently been proposed that hypoxia itself is a significant driver of thrombosis associated with COVID-19 (114). Prior studies have shown that the vascular response to hypoxia is controlled by hypoxia inducible transcription factors (HIFs), particularly at high altitudes. HIF-1α has recently been shown to play a critical role in the acute inflammatory response associated with acute respiratory distress syndrome (ARDS) associated with trauma and hemorrhagic shock (115). HIFs, including HIF-1α are known to promote thrombosis through plasminogen activator inhibitor (PAI) 1 and TF (116). Additionally, HIF-2 has a pro-thrombotic effect by inhibiting the action TF inhibitor (116). HTF-1α is expressed in alveolar epithelial cells and has been shown to trigger cellular inflammation with release of pro-inflammatory and potentially pro-thrombotic cytokines, including TNFα and interleukins such as IL-6 (103). Additionally, hypoxia is thought to have direct pro-thrombotic effects on the endothelium, including suppression of thrombomodulin and reduction of fibrinolytic potential (116). It has been theorized that targeting HIFs may ameliorate both COVID-19 induced endothelial damage and thrombosis (117). Further work is needed to determine the mechanisms by which hypoxia leads to thromboembolic events in COVID-19. Hypoxia induced transcription factors, activation of neutrophil extracellular traps, monocytes, complement, and pro-inflammatory cytokines are all potential contributors to “thromboinflammation” with COVID-19 and may represent pharmacologic targets for prevention treatment of thrombosis.

## COVID-19 Associated Coagulopathy: Recommendations for Prevention and Treatment

COVID-19 has presented the world with the unique challenge of discovering the basics of the pathophysiology and managing the disease at the same time. A number of societies have released guidance for the prevention and treatment of COVID-19 associated coagulopathy, while many groups have also released case studies detailing off label uses of pharmacologic agents based on clinical circumstance (118). The ISTH currently recommends that all hospitalized COVID-19 patients receive prophylactic dose low-molecular weight heparin in the absence of contraindications (4, 118). Other groups echo this guidance, and some state that consideration should be given to prophylaxis in high risk ambulatory patients as well as post-discharge patients with laboratory evidence of persistently elevated levels of D-dimer (62, 118). Studies have been performed on the safety of utilizing intermediate or therapeutic dose anticoagulation in a “prophylactic manner” for COVID-19 patients; however at this time, in the absence of documented thromboembolism, there is no conclusive guidance on using intermediate or therapeutic anticoagulation in COVID-19 (4, 119, 120). Tang et al. found that use of heparin for seven days was associated with a decrease in mortality in patients with a sepsis induced coagulopathy (SIC) score > 4, and a D-dimer level greater than six times the upper limit of normal (121). There is limited evidence that heparin may interact with the spike S1 protein domain on SARS COV-2 giving it both antiviral and anti-thrombotic properties (44, 122). It is recommended that anti-Xa be monitored to guide heparin treatment, rather than aPTT as discussed above due to the high prevalence of LAC and other factors making the aPTT unreliable in many COVID-19 patients (44).

Although institutional policies vary, many medical centers do not routinely screen for VTE, in the absence of clinical suspicion. Given the high burden of VTE seen in COVID-19 patients, questions regarding screening or empiric treatment with therapeutic anticoagulation have been proposed. D-dimer level has been shown to correspond to VTE risk in COVID-19 patients, as discussed above, with different screening thresholds proposed. Studies have advocated for VTE screening with bilateral lower extremity ultrasound for patients with a D-dimer level >2,000 ng/mL, and empiric treatment with therapeutic heparin for a D-dimer > 5,500 ng/mL, while others have shown a D-dimer level > 2,600 ng/mL to be predictive of VTE development, and still others who recommend therapeutic anticoagulation for a D-dimer level > 2,000 ng/mL on ICU admission or an increase greater than six times the admission level (53, 76, 123). Monitoring D-dimer levels may prove to be a helpful adjunct for clinical decision making regarding surveillance and treatment of VTE in COVID-19. With the high rates of VTE observed in patients receiving chemoprophylaxis, there is also discussion about the utility of alternate therapies, such as antiplatelet agents (124). In a single-center case control study, five critically ill COVID-19 patients were given an antiplatelet regimen in addition to heparin, with significant improvement in their pulmonary status as compared to controls (125). However, in a larger retrospective review of 3,772 hospitalized and ambulatory COVID-19 patients, 266 patients on antiplatelet agents at the time of admission were propensity matched to 798 patients not on antiplatelet agents or other anti-coagulants. Patients on antiplatelet agents were not found to have significantly different all-cause mortality (HR 1.029, CI 95% [0.723-1.466], p=0.997) or need for mechanical ventilation (HR 1.239, CI 95% [0.807-1.901], p=0.256) from those who were not on antiplatelet agents or other anti-coagulants (126). Use of anti-platelet agents specifically for the treatment of COVID-19 related coagulopathy is not currently recommended (127).

Fibrinolytic agents including tissue plasminogen activator (tPA), have also been used in a small number of cases. Under certain circumstances, off-label use without overt evidence of VTE has been advocated when it is not possible to obtain a CT PE study, or there is high clinical suspicion of PE with potential signs of PE on bedside echocardiogram (128). In a case series, Goyal et al. described administering low dose tPA to three patients on the verge of intubation due to the suspicion for pulmonary microthrombi, and all three were weaned from oxygen within 3-7 days (129). Several other small case series (between 3-5 patients) have described temporary clinical improvement in after administration of tPA, with descriptions of some patients being extubated and others avoiding intubation after tPA administration (130). However, these case series do not demonstrate a durable response to tPA in all patients, and as always the risks of systemic fibrinolysis, specifically devastating intracranial hemorrhage, need to be considered. Off label use of tPA in COVID-19 is not currently recommended, though it is being studied in an ongoing trial (NCT04357730).

Because of the high frequency of VTE in COVID-19 patients despite chemoprophylaxis, the question of need for greater than prophylactic doses of heparin has been raised (61, 69, 123, 131). Studies have demonstrated that these patients require higher doses of Unfractionated Heparin or Low Molecular Weight Heparin, titrated based on anti-Xa levels (57, 132). A recent review has advocated for using viscoelastic testing, such as TEG and ROTEM, to monitor efficacy of anticoagulant therapy in COVID-19 (133). Additionally, COVID-19 patients on long-term anticoagulation may receive some protective benefit, especially since many COVID-19 patients are admitted with VTEs from the ambulatory setting, suggesting the hypercoagulable phase of COVID-19 starts early in the disease process (123). In a cohort of 70 elderly COVID-19 patients in Italy (> 70 years old) being followed by an outpatient cardiology clinic, chronic direct oral anticoagulant (DOAC) use was independently associated with decreased mortality (134). However, other authors have not found a statistically significant mortality difference between patients on chronic anticoagulation and those who are not (126). Treatment with therapeutic anticoagulation in a “prophylactic” manner against COVID-19 associated coagulopathy is not currently recommended, though there is guidance towards considering chemoprophylaxis in the ambulatory or post-discharge setting if clinically appropriate, as it appears that the hypercoagulable state may persist beyond hospitalization in select patients. As more evidence becomes available, it is likely that a combination of clinical and laboratory factors will be needed to individualize treatment with anticoagulants, tPA, antiplatelet agents, or even recombinant thrombomodulin, in COVID-19 patients suffering thromboembolic events (133).

## Conclusion:

COVID-19 associated coagulopathy is a unique clinical and pathophysiologic challenge with a distinct laboratory phenotype that results from complex interactions between regulators of inflammation and coagulation. Resultant venous and arterial thrombosis both in macro- and microvasculature leads to significant morbidity and mortality in COVID-19. Many of the papers reviewed in this manuscript were case series, retrospective single and multi-center studies. It is imperative that ongoing clinical research is undertaken to determine pharmacologic strategies that will lead to improved outcomes. In particular, randomized controlled trials in COVID-19 patients with derangements in coagulation are needed to accurately risk-stratify patients for thrombotic complications and streamline treatment guidelines.

## Figures and Tables

**Table 1:** Summary of Relevant Lab Findings in COVID-19*

End point	Study	Country of Origin	Design	Centers	No. of COVID D-19 Patients	Relevant Findings
**D-dimer level**	Jin et al. (2)	China	Meta-analysis	Multi	4889	↑ D-dimer in non-survivors
	Tang et al. (3)	China	Retrospective	Single	183	↑ D-dimer in non-survivors
	Zhang et al. (10)	China	Retrospective	Single	343	↑ Mortality with D-dimer ≥ 2.0 μg/dL
	Yu et al. (11)	China	Retrospective	Single	1,561	D-dimer > 0.6 μg/mL on admission associated w/ severe COVID-19
	Chilmuri et al. (12)	USA	Retrospective	Single	375	D-dimer > 1,000 ng/mL on admission associated increased odds of in-hospital mortality
	Gayam et al. (13)	USA	Retrospective	Single	408	D-dimer is independent predictor of mortality in hospitalized African American patients
	Li Y et al. (14)	China	Prospective	Multi	279	Dynamic relationship between D-dimer and disease progression
	DiMinno et al (15)	Italy	Meta-analysis	Multi	1,511	↑ D-dimer in non-survivors
	Liu J et al. (16)	China	Retrospective	Single	1,190	↑ D-dimer on admission independent predictor of in-hospital death
	Li C et al. (17)	China	Retrospective	Single	749	Normal day 1 and day 3 D-dimer strongly associated with survival
	Artifoni et al. (19)	France	Retrospective	Multi	71	D-dimer level ≥ 3.0 μg/ml with 80% positive predictive value for VTE
	Cui et al. (20)	China	Retrospective	Single	81	↑ D-dimer in patients with VTE, ↑ D-dimer with anticoagulation
	Martin-Rojas et al. (21)	Spain	Retrospective	Single	206	↑ D-dimer independent predictor of thrombotic event
	Cho et al. (22)	USA	Retrospective	Single	158	D-dimer > 6,494 ng/mL associated with increased risk of DVT, NPV 88.0%
	Mestre-Gomez et al. (23)	Spain	Retrospective	Single	91	D-dimer ≥ 5000 ≥g/dL independent predictor of PE
	Demelo-Rodriguez et al. (24)	Spain	Prospective	Single	156	D-dimer ≥ 1570 ng/mL associated with DVT
**Platelet count**	Di Minno et al. (15)	Italy	Meta-Analysis	Multi	1,511	Thrombocytopenia associated with disease severity and mortality
	Liu Y et al. (28)	China	Retrospective	Single	383	Dose dependent association of platelet count with mortality
	Al-Samkari et al. (25)	USA	Retrospective	Multi	429	Admission ↑ platelets predictive of thrombotic complications Thrombocytopenia predictive of major bleeding event
	Chen W et al. (26)	China	Retrospective	Single	272	11.8% “delayed phase” thrombocytopenia with poor outcomes
	Yang et al. (29)	China	Retrospective	Single	1476	20.7% with thrombocytopenia, platelet nadir correlates to mortality
	Chen R et al. (30)	China	Retrospective	Multi	548	Lower platelet count on admission and ↓ trend through hospitalization in non-survivors
	Zhao et al. (31)	China	Retrospective	Single	532	Lower mean platelet count at multiple time points in non-survivors
	Hottz et al. (32)	Brazil	Prospective	Single	35	↑ Platelet activation in severe COVID-19
**VWF**	Goshua et al. (33)	USA	Retrospective	Single	68	↑ VWF activity in ICU and non-ICU
	Escher et al. (34)	Switzer land	Case series	Single	3	↑ VWF and Factor VIII with normal ADAMSTS13 and platelet count
	Blasi et al. (38)	Spain	Case series	Single	23	↑VWF and ↓ADAMSTS13 in COVID patients vs. healthy controls
**Anti-phospholipid antibodies**	Helms et al. (37)	France	Prospective	Multi	150	85% of tested with increased lupus anticoagulant
**Anti-phospholipid antibodies (cont)**	Xiao et al. (39)	China	Cross-section	Single	79	APLs seen in 47% of critically ill COVID-19 patients
	Bowles et al. (40)	UK	Prospective	Single	216	Lupus anticoagulant detected in 91% with prolonged aPTT
	Harzallah et al. (41)	France	Prospective	Single	56	Lupus anticoagulant (+) in 45%
	Siguret et al. (42)	France	Prospective	Single	74	85% with lupus anticoagulant, 12% with other APLs
	Galeano-Valle et al. (45)	Spain	Case series	Single	24	2 patients with weakly positive anti-cardiolipin and anti-B2-GP1 IgM.
**TEG/ROT EM**	Ibanez et al. (18)	Spain	Cross-section	Single	12	↑ Clot firmness on ROTEM
	Mortus et al. (48)	USA	Case series	Single	21	↑MA associated with thrombotic events
	Panigada et al. (49)	Italy	Cross-section	Single	24	↓R,K,Ly30; ↑MA in COVID-19
	Pavoni et al. (50)	Italy	Retrospective	Single	40	Hypercoagulable on ROTEM
	Wright et al. (51)	USA	Retrospective	Single	44	Fibrinolysis shutdown associated with VTE
	Creel-Bulos (52)	USA	Case series	Single	25	Fibrinolysis shutdown associated with thrombosis
	Yuriditsky et al. (53)	USA	Retrospective	Single	64	TEG not correlated to VTE

* Studies included are those which specify laboratory abnormalities seen in COVID-19 patients

**Table 2:** Clinical Findings of Venous and Arterial Thrombosis in COVID-19 Patients^Δ^

Endpoint	Study	Country of Origin	Design	Centers	No. of COVID -19 Patients	Frequency observed
**Overall thrombotic complications**	Klok et al.* (5)	Netherlands	Retrospective	Single	184	31%
	Fraisse et al.* (6)	France	Retrospective	Single	92	40%
	Al-Samkari et al. (25)	USA	Retrospective	Multi	400	9.5%
	Helms et al. (37)	France	Prospective	Multi	150	16.7%
	Lodigiani et al. (56)	Italy	Retrospective	Single	362	7.7%
	Beun et al. (57)	Netherlands	Retrospective	Single	75	33.3%
	Mayo Clinic unpublished	USA	Retrospective	Single	105	5.9%
**VTE**	Cui et.al. (20)	China	Case series	Single	81	25%
	Hippensteel et al.* (58)	USA	Retrospective	Single	91	26.1%
	Trimaille et al. (59)	France	Retrospective	Single	289	17%
	Chi et al. (60)	USA	Meta-analysis	Multi	1981	23.9%
	Hasan et al.* (61)	UK, Australia, Malaysia	Meta-analysis	Multi	899	31%
	Roberts et al. (62)	UK	Prospective	Multi	1877	4.5%
**Pulmonary Embolism**	Mestre-Gomez et al. (23)	Spain	Retrospective	Single	452	6.4%
	Bavaro et al. (63)	Italy	Case series	Single	20	40%
	Gervaise et al. (64)	France	Retrospective	Single	72	18%
	Whyte et al. (65)	UK	Retrospective	Single	1477	5.9%
	Fauvel et al. (66)	France	Retrospective	Multi	1240	8.3%
	Bompard et al. (67)	France	Retrospective	Multi	135	24%
	Poissy et al.* (68)	France	Case series	Single	106	20.6%
	Taccone et al.* (69)	Belgium	Retrospective	Single	40	33%
	Mak et al.* (70)	UK	Case series	Single	51	35%
**Deep Vein Thrombosis**	Demelo-Rodriguez et al.^†^ (24)	Spain	Prospective	Single	156	14.7%
	Chen Set al.^†^ (71)	China	Retrospective	Single	88	46%
	Zhang et al.*^†^ (72)	China	Retrospective	Single	143	46.1%
	Rieder et al. (73)	Germany	Prospective	Single	49	6.1%
	Santoliquido et al.^†^ (74)	Italy	Prospective	Single	84	11.9%
	Middeldorp et al.^†^ (75)	Netherlands	Prospective	Single	198	42% (at 21d)
	Trigonis et al.*(76)	USA	Retrospective	Single	45	42.2%
	Voicu et al.*^†^ (77)	France	Prospective	Single	56	46%
**Acute Cerebrovascular Disease**	Li Y et al. (79)	China	Retrospective	Single	219	4.6%
	Merkler et al. (80)	USA	Retrospective	Multi	2132	1.5%
	Yaghi et al. (82)	USA	Retrospective	Multi	3556	0.9%
	Tan et al. (84)	Singapore	Meta-analysis	Multi	4466	1.2%
	Rothstein et al. (85)	USA	Retrospective	Multi	844	2.4% ischemic 0.9% hemorrhagic

Δ Table includes studies that provide clinical outcomes

* Studies included patients admitted to ICUs only

† Studies utilized screening ultrasound for identification of DVT; except in Middeldorp et al. where only 55 of 198 patients underwent screening ultrasound.

**Table 3:** Summary of Findings Pertaining to Prevention, Treatment, and Diagnosis of Thromboembolic Events associated with COVID-19

Endpoint	Study	Country of Origin	Design	Centers	No. of COVID D-19 Patients	Relevant Findings
**Outpatient Anti coagulation**	Beun et al. (57)	Netherlands	Retrospective	Single	75	VTE patients required unusually high dose UFH > 35,000 IU/day for goal aPTT
	Taccone et al. (69)	Belgium	Retrospective	Single	40	High dose chemoprophylaxis associated with ↓ PE
	Roberts et al. (85)	UK	Retrospective	Single	1,877	↑ Post-discharge VTE (OR 1.6)
	Tang et al. (121)	China	Retrospective	Single	449	Lower mortality in patients with a SIC score ≥ 4 treated with heparin for ≥ 7 days
	Maatman et al. (123)	USA	Retrospective	Multi	109	Critically ill patients with D-dimer level > 2,600 ng/mL should get screening duplex
	Tremblay et al. (126)	USA	Retrospective	Multi	3,772	No difference in survival or time to mechanical ventilation with prior anticoagulant or anti-platelet
	White et al. (132)	UK	Retrospective	Single	69	↑ Heparin dosing needed to achieve therapeutic levels in 15 patients
	Rossi et al. (134)	Italy	Retrospective	Single	70	Chronic DOAC use independently predicts survival
**VTE Screening Thresholds**	Yuriditsky et al. (53)	USA	Retrospective	Single	64	Recommend therapeutic anticoagulation for ICU patients w/D-dimer level > 2,000 ng/mL or ↑ 6x-10x admission level
	Trigonis et al. (76)	USA	Retrospective	Single	45	Recommend ultrasound for D-dimer level > 2,000 ng/mL & consider therapeutic anticoagulation for D-dimer level > 5,500 ng/mL
**Use of Anti-Platelet Agents**	Russo et al. (124)	Italy	Retrospective	Multi	192	No change in ARDS or in-hospital mortality associated with anti-platelet or anticoagulation
	Viecca et al. (125)	Italy	Case Series	Single	5	Hypoxia improvement in ICU patients treated with specific anti-platelet regimen
**Use of Fibrinolytic Agents**	Bona et al. (128)	Italy	Case Series	Single	4	Clinical improvement in 3 of 4 patients treated with tPA for bedside diagnosis of PE
	Goyal et al. (129)	India	Case Series	Single	3	3 patients treated with tPA for respiratory failure weaned from oxygen within 3-7 days.
	Christie et al. (130)	USA	Case Series	Single	5	TPA given for worsening respiratory failure; improved respiratory status in all 5 patients

## Key Points:

- COVID-19 associated coagulopathy is characterized by a unique set of laboratory disturbances that are associated with poor prognosis, including elevated D-dimer level, thrombocytopenia, and hypercoagulable profile on TEG and ROTEM. Anti-phospholipid antibodies have also been proposed to play a role in COVID-19 related coagulopathy.
- Clinical manifestations of disrupted coagulation in COVID-19 patients include DVT, PE, acute ischemic stroke, and peripheral arterial thrombosis.
- Microvascular thrombosis has been observed in autopsy studies and likely as a result of “thromboinflammation”. Immune responses, including cytokine release, complement activation, NETosis, and monocyte-platelet aggregation, contribute to the pathophysiology of coagulopathy in COVID-19.
- Patients with COVID-19 infection, who require ICU care and have elevated D-dimer levels are at increased risk of developing thromboembolic complications.
- Vigilant monitoring of laboratory and clinical risk factors are warranted to guide chemoprophylaxis and treatment of thrombotic events for inpatients and post-discharge.
- Treatment with therapeutic anticoagulation in a “prophylactic” manner against COVID-19 associated coagulopathy is not currently recommended.
